# The influence and mechanism of gut microbiota on spermatogenesis

**DOI:** 10.1007/s10565-026-10168-1

**Published:** 2026-03-12

**Authors:** Xinxing Zhou, Zhongli Ge, Xinyue Yang, Yujuan Liu, Yaoyun Li, Luning Chen, Liqiong Lv, Guanghui Chen

**Affiliations:** 1 Department of Pharmacy, Renmin Hospital of Wuhan University, Wuhan, 430060 China; 2 ‌Lintong Rehabilitation and Convalescence Center, Xi’an, 710600 China; 3 Department of Neurosurgery, Rinmin Hospital of Wuhan University, Wuhan, 430060 China

**Keywords:** Spermatogenesis, Gut microbiota, Vitamins, Extracellular vesicles, Sphingolipid metabolites, Androgens

## Abstract

Gut microbiota (GM) serves several functions in host, including immunological modulation, maintaining intestinal epithelial cell barrier and defending against pathogen invasion. Previous research has demonstrated that GM could regulate distal organs such as gut-brain and gut-liver axis. Recently, a rising number of researchers have discovered the substantial link between GM alteration and spermatogenesis disorder in male. This review highlights the phenomena of sperm production abnormalities caused by GM dysbiosis and the putative molecular pathways. Microbiota dysbiosis could lead to abnormal sperm production through multiple pathways. For example, the metabolic disturbance of vitamins impairs meiosis and spermatogenesis, upregulated miR-211-5p of extracellular vesicles inhibits meiosis and damages spermatogenesis, abnormal sphingolipid metabolite affects spermatogenic cell apoptosis, decreased short-chain fatty acid level causes inflammation and damages testicular tissue and reduced androgen level, resulting in spermatogenesis disorder. In addition, this review highlights therapeutic strategies, such as the administration of vitamins, probiotics, and prebiotics, for the treatment of spermatogenesis disorders associated with GM dysregulation. These insights underscore the importance of targeting the GM to preserve sperm quality and improve male reproductive health, offering promising future directions for the treatment of spermatogenic dysfunction and infertility.

## Introduction

Spermatogenesis is the full process of primordial germ cells developing into spermatogonocytes and becoming mature sperm(Du et al. 2021). Throughout spermatogenesis process, Sertoli cells surround germ cells and supply the required nutrients, androgens released by Leydig cells could diffuse spermatogenic tubules and promote sperm production and maturation(Hess and Renato Franca 2008; Walker 2021). Spermatogenesis is a strictly regulated process and the abnormality of any procedures may lead to spermatogenesis disorder, manifested as the reduction of sperm quantity and quality(You et al. 2021). In addition, male infertility is closely related to spermatogenesis disorder, the current global incidence of which is about 10% to 15%(Kanem 2023; Oud et al. 2022). Recently, the studies have shown that sperm function is affected by inflammatory bowel disease(Darmadi et al. 2023; Wdowiak et al. 2021). Therefore, the disturbance of gut microbiota (GM) may be a key reason for spermatogenesis disorder.

GM is a crucial symbiotic system in the human body, playing a key role in maintaining health by regulating immunity, preserving the intestinal barrier and protecting against pathogens(Wang et al. 2024; Yang et al. 2020; Zhang 2022). It consists of approximately 10^14^ microorganisms, including bacteria, fungi, archaea and viruses(Guo et al. 2022). These microbes are classified into seven major phyla, including Firmicutes, Bacteroidetes, Actinobacteria, Proteobacteria, Verrucomicrobia, Cyanobacteria and Clostridia(Lucas et al. 2021). Bacteroidetes (such as Bacteroides and Prevotella) are typically beneficial but may become opportunistic pathogens under dysbiosis(Wang et al. 2021). Firmicutes (such as Lactobacillus) contribute to health by producing short-chain fatty acids (SCFAs) and inhibiting pathogens(Sun et al. 2023). Proteobacteria (such as E. coli and Salmonella) are linking to inflammation(Sharma et al. 2022; Sun et al. 2021). Additionally, beneficial genera (such as Bifidobacterium) may improve metabolic and immune functions(Könönen and Wade 2015). Ultimately, the diversity and stability of GM are necessary for host physiology(Wang et al. 2024; Yang et al. 2020).

More and more studies have demonstrated that GM affects the function of distal organs via diverse mechanisms such as gut-brain and gut-liver axis(Góralczyk-Bińkowska et al. 2022; Tilg et al. 2022). The gut-brain axis is the subject of extensive investigation. For instance, the central nervous system disorder (such as depression, anxiety and autism) are associated with the dysregulation of GM(Góralczyk-Bińkowska et al. 2022; Mayer et al. 2022; Wang et al. 2023a). Moreover, GM can regulate the metabolic processes of the liver and influence the onset of early-stage liver diseases such as alcoholic liver disease and non-alcoholic fatty liver disease(Liu et al. 2025; Wang et al. 2025). Recent research has established the pivotal role of GM on spermatogenesis through the newly proposed gut-germ cell axis(Argaw-Denboba et al. 2024). This axis operates via multiple interconnected mechanisms. GM dysbiosis triggers systemic inflammation by elevating pro-inflammatory cytokines (such as interleukin-6 and tumor necrosis factor-α) to impair blood-testis barrier and germ cell development(Ding et al. 2020; Wang et al. 2023b). Concurrently, microbial metabolites like SCFAs regulate Leydig cells function and testosterone synthesis for influencing spermatogenesis(Rato et al. 2012). Together, these findings underscore the gut-germ cell axis as a critical interface between microbial ecology and male reproductive health. This review comprehensively summarizes the relevant research advancements that GM mediates spermatogenesis disorder. It primarily focuses on summarizing the regulatory mechanisms underlying spermatogenesis disorders and the potential gut microbiota-based intervention strategies.

## Factors causing GM disturbance

GM is usually altered by external environmental factors. For instance, delivery mode, diet, occupational environment, and antibiotic use can influence its diversity.

### Delivery mode

Delivery mode stands as a pivotal factor affecting the colonization of gastrointestinal flora in neonates. Studies have shown that the GM composition of vaginally delivered babies is similar to the maternal vaginal microbiota(Ma et al. 2023). In these infants, anaerobic bacteria (such as Lactobacillus and Prevotella) are the dominant group. In contrast, cesarean delivery has the potential to induce an imbalance in the infant's gut flora and reduces its diversity(Ma et al. 2023). On one hand, infants delivered by cesarean section lack access to the maternal vaginal microbiome. On the other hand, the hospital environment and maternal skin become the primary ways for infants to contact the outside world. Consequently, hospital pathogens (such as Enterococcus, Enterobacter and Klebsiella) dominate the infant's gut(Ma et al. 2023). The appropriate addition of probiotics, prebiotics and synthetic bacteria could promote the optimal colonization of GM in baby's body(Martín-Peláez et al. 2022).

### Diet

Diet is one of the critical factors affecting the diversity and abundance of GM. Prolonged consumption of a high-fat diet could affect the abundance of GM. This phenomenon could be attributed to the fact that the nutrients for GM predominantly originate from carbohydrates that remain undigested and unabsorbed by small intestine(Attene-Ramos et al. 2010). Furthermore, lipid metabolites generated from a high-fat diet (such as secondary cholic acid and hydrogen sulfide) could damage intestine mucosa, trigger mucosal inflammation and alter the GM microenvironment(Resta 2009). Specifically, the abundance of beneficial bacteria (such as Lactobacillus and Bifidobacterium) was decreased, while the level of pathogenic bacteria (Bacteroides and Fusobacterium) was increased in high-fat diet-fed mice(Ding et al. 2020; La Serre et al. 2010). In addition, vitamins also influence the composition of GM. For example, vitamin A promotes the growth and differentiation of intestinal cells to maintain intestinal barrier function(Pham et al. 2021). Vitamin B2 deficiency increases Prevotella abundance and decreases Bacteroides abundance(Carrothers et al. 2015). Vitamin K contributes to increasing the level of Lactobacillus and Bifidobacterium in the gut(Seura et al. 2017). To sum up, the composition of GM is easily affected by diet.

### Environmental Factors

Occupational exposure to chemical, physical and biological hazards in workplace constitutes a significant factor affecting the composition of microbiome(Fenga 2022). Differences in microbial composition could be used as biomarkers to diagnose and monitor workers' health(Mucci et al. 2022). Beyond occupational exposure, environmental pollutants generated by human activities, agriculture and industry could also affect GM, the primary pollutants encompass heavy metals, pesticides and polychlorinated biphenyls(Lu et al. 2015). For example, the heavy metal mercury could increase the level of actinomyces and proteus and reduce the number of Bacteroides in intestine(Lapanje et al. 2010). Pesticides could lead to decreasing beneficial bacteria level (such as Lactobacillus, Bifidobacterium and Enterococcus) in gut(Joly et al. 2013). Polychlorinated biphenyls could reduce the overall abundance of GM, especially for Proteobacteria(Choi et al. 2013). These pollutants affect human health by altering the composition of GM.

### Antibiotics

Antibiotics, which are chemicals produced by microorganisms, plants and animals, frequently used to eliminate or prevent the colonization of bacteria in body for preventing and treating various bacterial infections. Nevertheless, their utilization could significantly affect the composition of GM and reduce its abundance for lacking of specificity towards particular bacterial types(Ainonen et al. 2022). Different antibiotics have different effects on GM. For instance, the number of gram-positive bacteria increases while anaerobic bacteria remains unaffected after the administration of narrow-spectrum anti-aerobic drug amtraxam, the intestinal microflora returns to the normal level after drug withdrawal(Saene et al. 1998).The numbers of anaerobic bacteria, gram-negative bacteria and gram-positive bacteria are decreased significantly when the narrow-spectrum anti anaerobic drug clindamycin is used(Saene et al. 1998). The use of the broad-spectrum antibiotic (such as cefoperazone) also causes a general decline in the number of aerobic and anaerobic bacteria(Saene et al. 1998). Antibiotics (neomycin 2.5 mg/ml, bacitracin 2.5 mg/ml and pimaricin 1.25 ug/ml) could alter DNA methylation patterns of sperm in paternal mice, cause developmental delay and reduce survival rates of offspring by disrupting GM(Argaw-Denboba and Schmidt 2024). As different antibiotics possess distinct antibacterial spectra and different effects on GM, we must make rational selections of antibiotics to minimize the risk of disturbing GM.

## The regulatory mechanism between GM and spermatogenesis

The GM functions mainly depend on its small metabolites. Here, we focused on the effect of these small molecules (including vitamins, EVs, sphingolipid metabolite, SCFAs and androgens) on spermatogenesis.

### Vitamin

**Fig. 1:**
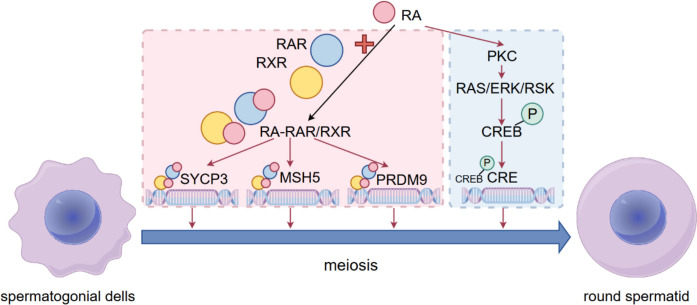
Mechanism of action of RA in sperm meiosis. RA regulates sperm meiosis mainly through the following two ways. First, retinoic acid (RA) binds to retinoic acid receptor (RAR) or retinoid X receptor (RXR) in sperm cells to form a complex that promotes the expression of synaptonemal complex protein 3 (SYCP3), MutS homolog 5 (MSH5) and PR domain-containing protein 9 (PRDM9). In addition, retinoic acid can activate protein kinase C (PKC) and phosphorylate cAMP-response element binding protein (CREB) through rat sarcoma virus/extracellular signal-regulated kinase/ribosomal S6 kinase (RAS/ERK/RSK) pathways. Phosphorylated CREB could activate the expression of CRE gene and promote the meiosis process

The study have suggested that vitamins could regulate the functions of Sertoli cells, Leydig cells and sperm to facilitate spermatogenesis(Jueraitetibaike et al. 2019; Blomberg Jensen 2014; Wu et al. 2024). The absorption process of vitamin A (a fat-soluble vitamin) is intricately linked to the metabolism of bile acids. Bile acids consist of primary bile acids and secondary bile acids, synthesized by liver and GM, respectively(Hamilton et al. 2007). Bacterial metabolism requires bile brine hydrolysis enzyme (commonly discovered in Bacteroidetes, Firmicutes and actinobacteria) to hydrolyze amino acids(Guzior and Quinn 2021). The GM could facilitate the absorption of vitamin A by producing secondary bile acids(Guo et al. 2022). Secondly, E. coli could produce retinoic acid receptor protein to promote the transport of vitamin A to intestinal cells. Additionally, GM can prevent the degradation of vitamin A by inhibiting the activity of cytochrome P450 family proteins(Stacchiotti et al. 2021). Vitamin A (an indispensable nutrient for reproductive system and embryonic development) could activate the molecular pathway that governs the differentiation of spermatogonia into mature sperm(Gurel et al. 2023; Zhang et al. 2022). Retinoic acid (RA, a metabolite of Vitamin A) is able to regulate testicular signal transduction and affect sperm differentiation and meiosis (the detailed process was shown in Fig. 1)(Zhou and Wang 2022). RA activates key genes involved in meiosis through its receptors RA receptors and retinoid X receptors such as MutS homolog 5, PR domain-containing protein 9 and synaptonemal complex protein 3 at late meiotic stages in spermatogenic cells(Wang et al. 2016). MutS homolog 5 and synaptonemal complex protein 3 involve in chromosome pairing and recombination, while PR domain-containing protein 9 is related to chromosome recombination(Marín-García et al. 2024; Zhang et al. 2023). RA could activate protein kinase C and transmit the activated signal to cyclic adenosine monophosphate (cAMP) response element-binding protein through sarcoma/extracellular signal-regulated kinase/ribosomal S6 kinase pathway(Hong et al. 2008). Phosphorylated cAMP response element-binding protein could regulate the expression of downstream genes for spermatogenesis (Deng et al. 2017). Zhang et al. demonstrated intestinal flora disturbance could impair spermatogenesis and disrupt vitamin metabolism in metabolic syndrome sheep model, mediated by decreasing level of Ruminococcaceae abundance and secondary bile acids through 16S rRNA sequencing and metabolomic analysis(Zhang et al. 2022). In addition, heat stress-induced dysbiosis of the gut microbiota impairs spermatogenesis by regulating secondary bile acid metabolism in the gut of mice(He et al. 2024).

Vitamin B9 (commonly as folic acid) plays a pivotal role in spermatogenesis. Folic acid is essential for the synthesis of DNA and RNA. Folic acid helps germ cells to resist oxidative stress and inflammation for preventing DNA damage and apoptosis and protecting the proliferation and differentiation of germ cells(Rad et al. 2021). Metagenomic analysis results show that folate acid is synthesized and metabolized by Lactobacillus and bifidobacteria(Rad et al. 2021). In addition, Kadry et al. found that lactobacillus and bifidobacterium supplement could counteract cadmium chloride-induced testis toxicity and sperm quality decline(Kadry and Megeed 2018). To sum up, vitamin B9 may affect spermatogenesis through intestinal flora. Unfortunately, no studies have directly confirmed that intestinal flora affects spermatogenesis through vitamin B9. Therefore, this blank needs to be confirmed by further research. Future studies could employ multi-omics integrated analysis to investigate dysregulation of vitamin B9-related metabolic pathways in GM dysbiosis models and correlate with the phenotypes of spermatogenesis impairment (such as spermatogonial differentiation arrest and meiotic abnormalities). Additionally, a co-culture system in vitro could be designed to incubate spermatogenic precursor cells with microbial metabolites (such as folate derivatives) and assess their effects on the expression of meiosis-related key genes (such as stimulated by retinoic acid gene 8 and synaptonemal complex protein). Finally, clinical cohort studies could analyze the associations between GM composition, serum/seminal folate levels and semen parameters in infertile men to provide translational evidence for mechanistic research.

It's worth noting that mouse models are critical for elucidating the major pathways responsible for bile acid biosynthesis and metabolism. Among the challenges faced by attempts to use the mouse models to study the role of bile acids in the pathogenesis of human are important species differences(Li and Dawson 2019). Humans and mice have substantially different bile acid pool compositions, such as the synthesis of ursodeoxycholic acid, hydroxylation at the 6-position of the bile acid steroid nucleus to generate muricholic acid, and the specificity of bile acid N-acyl-amidation(Sayin et al. 2013; Takahashi et al. 2016; Hofmann et al. 2010). Therefore, the GM-vitamin A-germ cell axis may differ between mice and humans due to the crucial role of bile acids in vitamin absorption.

### Extracellular vesicles

EVs are employed as carriers for intercellular communication. These vesicles are transported to distal cells and exert their functions via bloodstream and through endocytosis, respectively(Isaac et al. 2021). Lots of studies have demonstrated that EVs could regulate sperm maturation(Machtinger et al. 2016; Rowlison et al. 2020). In addition, microbiota-derived EVs could enter circulation and distal tissue like the liver and reproductive organs by crossing intestinal barriers(Zhao et al. 2022; Chen et al. 2024a). Recently, a study proposed the concept of the GM-EVs-RA-testicular axis and demonstrated that depletion of the GM caused changes in the function of EVs, which led to abnormal RA metabolism in testis, thereby impairing meiosis and spermatogenesis processes(Chen et al. 2024b). In addition, it is the dysbiosis of GM, which leads to abnormal upregulation of miR-211-5p in gut-derived circulating EVs, which inhibited the expression of meiosis-specific with coiled-coil domain in spermatocyte and impaired spermatogenesis by disturbing meiosis process(Chen et al. 2024a). Therefore, GM disturbance could affect the expression of small RNA in EVs and interfere with spermatogenesis.

### Sphingolipid metabolites

Sphingolipid is a principal constituent of cell membranes and undergoes degradation into ceramide, sphingosine and sphingosine 1-phosphate by alkaline sphingomyelase and neutral ceramidase in gastrointestinal mucosa(Sukocheva et al. 2020). Additionally, the metabolism of sphingolipids is influenced by GM. Currently, Bacteroides fragilis has been demonstrated to generate three distinct types of sphingolipid metabolites: ceramide phosphoethanolamine, dihydroceramide base and sphingolipid α-galactosylceramide(Wieland Brown et al. 2013). Fecal microbiota transplantation from prediabetic sheep induced elevating sphingosine level and impairing spermatogenesis in recipient mice(Sun et al. 2022). Hence, GM disturbance could cause spermatogenesis disorder by affecting the metabolism of sphingolipids.

### Short-chain fatty acid

SCFAs refer to organic fatty acids, primarily encompassing acetate, propionate and butyrate. In gut, acetate and propionate are predominantly produced by Bacteroides, while butyrate is produced by Firmicutes(Louis and Flint 2017). SCFAs could perform a variety of functions for regulating the host immune response and barrier function(Tan et al. 2023; Yao et al. 2022; Zhu et al. 2022). Simultaneously, SCFAs could also promote the production of mucin and the release of antimicrobial peptides(Martin-Gallausiaux et al. 2021). Mucin could bind to the lectin-like protein on immune cells and restrain the inflammatory response(Hamer et al. 2008). Antimicrobial peptides work with other mediators in host defense and produce innate immune responses and adaptations to infectious agents(Bin Hafeez et al. 2021; Erdem Büyükkiraz and Kesmen 2022). The testis is an immune-privileged organ. Its seminiferous tubules are enveloped by a basement membrane, which is structurally composed of Sertoli cells, intercellular tight junctions and myoid tubular cells(Qu et al. 2020). Male haploid germ cells, which express unique immunogenic autoantigens, are protected from immune attack by the blood-testis barrier(Wang et al. 2017). Therefore, maintaining immunosuppression of testicular environment is essential for spermatogenesis. Dysbiosis of gut microbiome aggravated male infertility in captivity by increasing SCFAs influenced the immune response in plateau pikas(Zhang and Tang 2024). The mixed probiotics could ameliorate the disorder of testicular dysfunction and spermatogenesis by restoring balance of SCFAs(Wu et al. 2024). In summary, the stability of GM is conducive to reducing the occurrence of testicular inflammation and maintaining spermatogenic environment by regulating the production of SCFAs.

### Androgens

Androgens is a kind of neuroactive steroid hormones and synthesized by testis and adrenal glands, which play a pivotal role in the growth and development of male. They are responsible for facilitating the formation and maintenance of secondary sexual characteristics, as well as initiating and sustaining spermatogenesis(Alemany 2022). In addition, androgens also affect the immune system and other physiological functions. Androgens suppress natural killer cell cytotoxicity, mitigate the occurrence of immune stress and protect the integrity of blood-testis barrier(Gubbels Bupp and Jorgensen 2018; Tang et al. 2022; Meng et al. 2005). Androgens are metabolized by liver and excreted into the intestine through bile, approximately 80% of which are reabsorbed into bloodstream(Schiffer et al. 2018). It has recently been reported that gut microbes could influence steroid hormones level. Firstly, GM could secrete β-glucuronidase and promote the hydrolysis of glucose-acidified testosterone and dihydrotestosterone and the reabsorption of free testosterone through the intestine(d'Afflitto et al. 2022). Secondly, Clostridium, Ruminococcus and other strains could convert glucocorticoids, pregnenolone and hydroxypregnenolone into androgens(Ridlon et al. 2013). Again, Lactobacillus and Clostridium intestinalis (ATCC 35704) have been discovered to express steroid-processing enzymes and produce steroid hormones to boost testosterone and dihydrotestosterone level(Diviccaro et al. 2020). Furthermore, GM could synthesize Vitamin K2 to improve the anti-inflammatory capacity of testis and increase the expression of steroid hormone synthase gene (such as Cytochrome P450 Family 11 Subfamily A Member 1) and blood testosterone level(Paulus et al. 2024). The bacteria expresses 3β-hydroxy steroid dehydrogenase (such as Mycobacterium neoaureum) to degrade androgens into androstenediones for reducing androgen level in depression men patients(Li et al. 2022). In conclusion, the intestinal flora could promote the generation and metabolism of androgens through multiple ways for maintaining testosterone level and dihydrotestosterone in testis microenvironment.

## Treatments of spermatogenesis disorder caused by intestinal flora disorder

Based on the regulation mechanism of intestinal flora on spermatogenesis, lots of new therapies have been proposed to treat spermatogenesis disorder. Here, we focus on vitamins, probiotics and prebiotics.

### Vitamin

RA is used for maintaining male reproductive tract and spermatogenesis. The study has demonstrated that spermatogenesis is arrested at spermatogonial stage in vitamin A-deficient mice(Pelt and Rooij 1990). Spermatogonocytes could be stimulated to resume differentiation and spermatogenesis disorder could be restored after supplementing vitamin A(Hogarth and Griswold 2010). Meanwhile, supplemental RA is used for the treatment of cryptorchidism and spermatogenesis disorder caused by varicocele(Peng et al. 2016; Perrotta et al. 2015). RA holds significant advantages in future clinical treatment domain of male reproductive dysfunction for the peculiarity of low cost and low toxicity. Most of male fertility supplements contain folic acid to provide a carbon source for DNA synthesis and methylation in sperm for scavenging free radicals and promoting spermatogenesis(Wong et al. 2002). Nevertheless, as reported by JAMA, a multitude of clinical trials have indicated that the use of folic acid in men does not lead to a significant improvement in semen quality(Schisterman et al. 2020). This phenomenon may be attributed to factors such as insufficient sample size in these trials or individual variances in the absorption, utilization and metabolism of folic acid.

### Probiotics and Prebiotics

Probiotics are living microorganisms that could promote human health and treat a variety of diseases. Lactobacillus and bifidobacterium are widely used probiotics(Mazziotta et al. 2023). They are able to inhibit pathogen growth, improve intestinal barrier function and regulate immune system(Mazziotta et al. 2023; Kocot et al. 2022; Piatek et al. 2020; Rousseaux et al. 2007; Wu et al. 2022). Previous research found that probiotics supplementation improved sperm quality and reduced the level of fragmented DNA, intracellular H_2_O_2_ and intracellular oxidative stress(Abbasi et al. 2021; Valcarce et al. 2017). Prebiotics are a type of dietary supplement, including oligosaccharides, galactooligosaccharides and breast milk oligosaccharides(Swanson et al. 2020). Prebiotics could increase the level of certain probiotics (such as bifidobacterium and Lactobacillus) and SCFAs to perform immune regulation(Valdes et al. 2018). Studies have shown that oral probiotics and prebiotics could improve sperm quality in patients with asthenospermia(Rodrigues et al. 2021). Therefore, these evidence suggests that probiotic and prebiotic supplementation may contribute to improving sperm quality.

## Conclusion and prospect

**Fig. 2:**
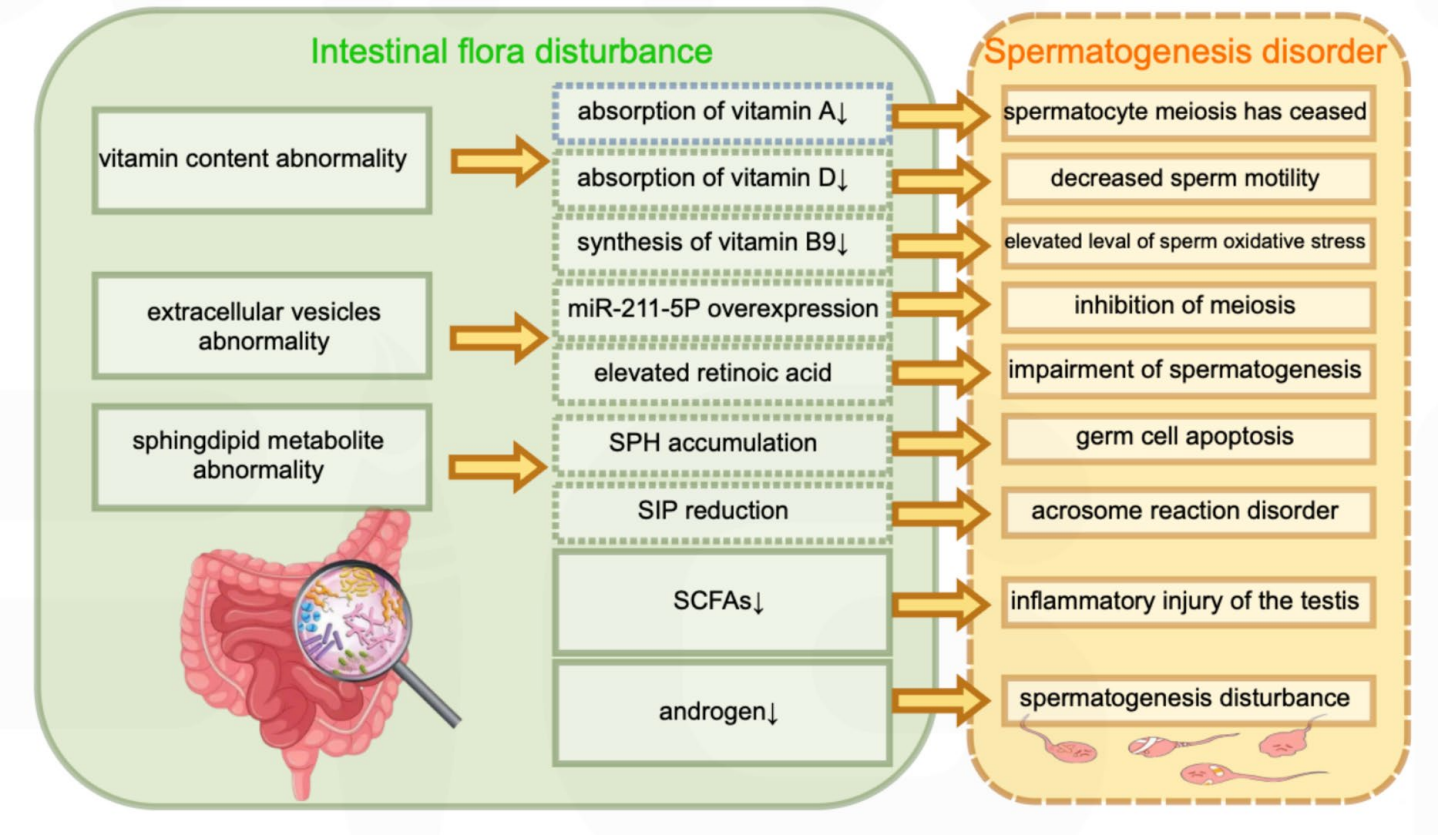
Overview of the mechanism of action between intestinal flora disturbance and spermatogenesis disturbance. SPH, sphingosine; S1P, sphingosine 1-phosphate; SCFAs, short-chain fatty acids

Recently, the relationship between GM and spermatogenesis has increasingly captured the attention of scholars. A recent landmark study demonstrated that paternal microbiome perturbations could directly impact offspring fitness through gut-germ cell axis(Argaw-Denboba et al. 2024). This review summarizes relevant research and progress on gut-germ cell axis, focusing on the regulation mechanism of GM on spermatogenesis by affecting the metabolism of vitamins, EVs, sphingolipid metabolites, SCFAs and androgens (Fig. 2). These findings provide valuable insights into male reproductive health. However, certain limitations must be considered. First, many conclusions are derived from animal studies (such as mice and sheep), key physiological differences between species may limit clinical translation and applicability. Second, the gut-germ cell axis is a nascent field with limited human data, therefore, further clinical research and GM-target interventions (such as probiotics and fecal microbiota transplantation) should be conducted to validate the potential mechanism of spermatogenesis disorder caused by the disturbed GM. Meanwhile, future research should integrate multi-omics approaches to identify conserving pathways and biomarkers from huge data, ultimately bridging the gap between preclinical findings and clinical applications in male infertility. Addressing these questions will be critical for harnessing GM's therapeutic potential and improving outcomes in male infertility.

## Data Availability

Not applicable.

## Abbreviations

EVs: Extracellular vesicles
GM: Gut microbiota
RA: Retinoic acid
SCFAs: Short-chain fatty acids
cAMP: Cyclic adenosine monophosphate
